# Challenges, experiences, and potential supports for East and Southeast Asian mothers in the workforce: a systematic review

**DOI:** 10.1186/s12905-024-03255-0

**Published:** 2024-07-25

**Authors:** Peh Joo Ho, Tomiko Mei Ying Sim, Christine Kim Yan Loo, Jingmei Li

**Affiliations:** Genome Institute of Singapore (GIS), Agency for Science, Technology and Research (A*STAR), 60 Biopolis Street, Genome, Singapore, 138672 Republic of Singapore

**Keywords:** Transition back to work, Re-entry, Working mothers, Asia

## Abstract

**Objective:**

To examine the challenges faced by Asian working mothers with a focus on re-entry to the workplace. In addition, we highlight potential supports that retain women in the workforce.

**Design:**

A systematic review was conducted according to the Preferred Reporting Items for Systematic Reviews and Meta-Analyses (PRISMA) recommendations and registered with PROSPERO database (CRD42022341130).

**Methods:**

Three independent reviewers were involved in the study selection to screen the search results sequentially by title, abstract, and full text using predefined inclusion and exclusion criteria. The methodological quality of each article was assessed via the Critical Appraisal Skills Programme (CASP) tool.

**Results:**

We analysed a total of 36 studies conducted across different regions: 30 from the East and 6 from Southeast Asia. Among these studies, 20 were quantitative in nature, 15 were qualitative, and one intervention. The 36 studies cover five themes: 1) policies, 2) external support sources, 3) external pressure, 4) breastfeeding and 5) health status. Within each theme the same factor can have a positive or negative impact on the mother depending on her having a pro-career or pro-family mindset.

Companies can take various initiatives to support working mothers, such as providing facilities for expressing breast milk at the workplace, educating staff to promote breastfeeding and accommodate childcare needs, and extending maternity leaves. However, there is a lack of literature that directly addresses the barriers and concrete support available to working mothers in Asia, beyond the scope of breastfeeding.

**Conclusions:**

Our findings underscore several obstacles that can impede a woman's seamless return to work. Pro-family and pro-career mothers have differing needs that cannot be addressed at the same time. There is a lack of comprehensive understanding regarding effective strategies or interventions that can support a positive reintegration into the workforce.

**Supplementary Information:**

The online version contains supplementary material available at 10.1186/s12905-024-03255-0.

## Introduction

The workforce today comprises a significant number of working mothers [1]. However, according to the 2019 global estimates from the International Labour Organization, mothers with partners and at least one child under the age of 6 years at home had a labour force participation rate of 55%, which was lower compared to the overall participation rate for women (62.1%) and significantly lower than that of fathers (97.1%). In fact, East and Southeast Asian mothers in households with partners and small children are observed to have significantly lower participation rates than that of European, Northern American, Australian, New Zealand and Sub-Saharan African working mothers from 2010 to 2020 [2]. This contrast is due to the common expectation of women being married and bearing children, influenced by deep-rooted cultural and traditional Confucian values [3]. As a result, these responsibilities frequently lead mothers to devote their time on early childcare.

Even as these cultural values evolve and an increasing proportion of Asian women enter the workforce, the primary role of childcare remains mainly a responsibility for mothers [4]. This requirement to juggle both career and family places immense stress on working mothers [5]. Subsequently, working mothers find themselves at different points on the spectrum of priorities, ranging from being pro-career to pro-family. With varying priorities, working mothers make different choices at the workplace and at home, leading to a range of experiences and challenges.

Existing literature highlights the general challenges faced by working mothers, such as the issue of women “opting out” to fulfill familial and child-rearing responsibilities and being “blocked out” of re-entry into the labour market due to a perceived lack of skillset [6]. It has also been raised that working mothers suffer a "motherhood penalty", which manifests as disadvantages in areas such as pay, perceived competence, and benefits that working mothers receive in the workplace compared to childless women [7–10]. Current research studies are often independent and have a generalized outlook, which prevents them from capturing the nuanced experiences of mothers in the workforce. Our study acknowledges this by not treating working mothers as a homogenous group and highlighting the discrepancies in their needs and priorities.

Considering the decline of birth rates in East and Southeast Asia [11, 12], it is evident that striking a balance between career and motherhood is becoming an increasingly demanding task. Therefore, exploration of this topic can have implications for both the wellbeing of working mothers and efforts to boost natality rates in region [13]. The systematic review aims to (1) fill the mentioned gaps in the existing literature by taking an Asian (East and Southeast) perspective, (2) examine the challenges reported by working mothers with a focus on re-entry to work, and (3) highlight solutions that inform decision-making on initiatives to retain women in the workforce.

## Methods

### Protocol and registration

The Preferred Reporting Items for Systematic Reviews and Meta-Analyses (PRISMA) guideline was used to guide the reporting of this systematic review. The protocol was prospectively registered with PROSPERO (International Prospective Register of Systematic Reviews) at the Genome Institute of Singapore as CRD42022341130.

### Search strategy

**Fig. 1:**
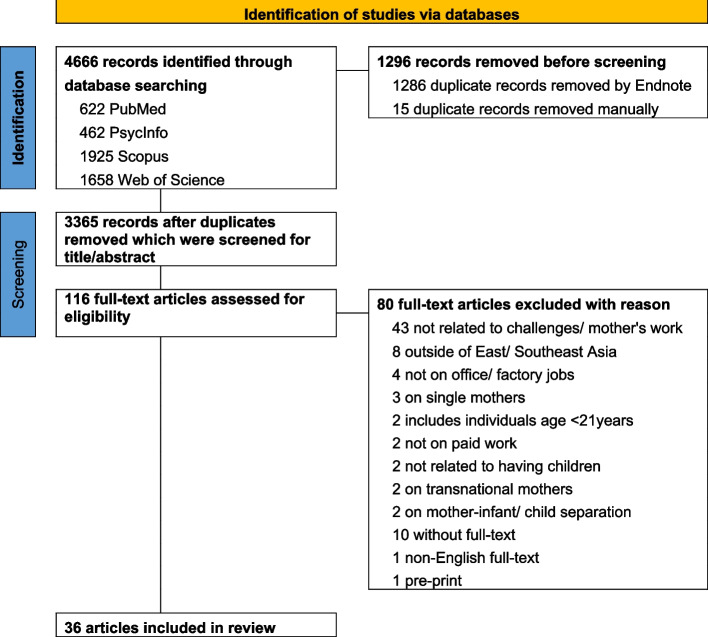
Flow diagram of study selection

On 31 May 2022, we collected data required for the systematic review from PubMed, PsycInfo, Scopus, and Web of Science databases. In addressing the challenges faced by working mothers in the workplace and in re-entry from the Asian perspective, we chose key search terms with Boolean operators. Terms such as “mothers”, “return to work”, “career”, “re-entry”, “postpartum” and “Asia” were used. The complete search strategies are available in Supplementary Table 1. After removing duplicates, three independent reviewers (TMYS, PJH, CKYL) screened the articles using a set of eligibility criteria by title and abstract, and subsequently the full texts (Fig. 1). Cross-referencing was then done to identify additional studies. Any discrepancies between reviewers were resolved through discussion. Unresolved disagreements were referred to a third investigator (JL) for review and resolution.

### Eligibility criteria

Our inclusion criteria encompassed full-text studies that focused on non-transnational mothers of working age (between 18 and 65 years) residing in East or Southeast Asia. The studies considered were specifically those involving paid employed/ self-employed mothers, and the publication date was limited to those released after the year 2010. The rationale behind limiting the publication date is to ensure that we analyse current mindsets and challenges faced by working mothers that is considered relevant to the present. We excluded articles on women younger than 18 years; listed as adolescent pregnancy; studies on single mothers or mother-infant separation; studies exclusively on professional athletes, performers or factory-workers; published before 2010; were not written in English; or were systematic reviews, meta-analyses, or pilot studies. Although the problems faced by working mothers vary by industry, it was observed that those of professional athletes or performers were drastically different from the majority population of working women in East and Southeast Asia, which does not align with the scope of our systematic review.

### Data extraction

Information describing the study design and population were extracted:

- Study characteristics, such as the country, year of study, and study design.
- Demographics of the study participants, including age, ethnicity, and employment.

For qualitative studies, information was extracted as quotes by the participants or findings expressed by the author in the absence of quotes. Association statistics, such as correlations and relative risks, to examine relationships between variables were extracted from quantitative studies. As the quantitative studies were varied in study design and factors studied, no fixed statistics was selected for extraction.

Variables associated with challenges (e.g. inadequate facilities, lack of support from co-workers, and mental health) were extracted to examine the challenges and experiences faced by working mothers (aim 2). Broadly, we extracted information in three categories (societal, workplace or personal) to highlight solutions that inform decision-making on initiatives to retain women in the workforce (aim 3) – e.g. workplace policies, co-worker and familiar support sources, and available facilities.

### Integrative review

In the synthesis of study findings, we used the integrative review methodology [14]. The results from our search were tabulated, extracted and further sorted into the theme clusters. This is also known as data reduction, which formulates an overall classification system that manages data from diverse methodologies. The rationale of this step is to organise data from primary sources into a framework that is manageable for analysis.

### Quality assessment

The evaluation of qualitative studies was conducted using the Critical Appraisal Skills Programme (CASP) tool (accessed date: 31 May 2022), which primarily focuses on aspects of validity, results, and relevance [15]. This tool is widely accessible and commonly utilized in the field. It consists of six questions addressing the validity of the study, three questions pertaining to the study's findings, and one question concerning the generalizability of the study.

The evaluation of quantitative cohort and cross-sectional studies involved the utilization of the respective scales from the Newcastle–Ottawa Quality Assessment Scale [16]. The scales encompass four questions related to the study's sample, one question regarding comparability, and two questions concerning the study's outcomes.

### Public involvement

The public was not involved in the development of the research question or the design and conduct of this systematic review.

## Results

On January 9, 2024, our search strategy produced a total of 4,666 articles. Three reviewers conducted an independent screening of 3,365 unique articles based on their title and abstract (Fig. 1). Subsequently, the full text of 116 articles was thoroughly examined, resulting in the inclusion of 36 articles for this study (Tables 1 and 2).

**Table 1:** Description of cohort, cross-sectional, and intervention studies

Author, Year of Study	Region	Country	Ethnicity	Study Design	Study period	Sample size	Selection and specification of study sample	Age, years
Yoshihiro Miyake, 2011 [17]	East	Japan	Japanese	Cohort	November 2001 to March 2003	1002 baseline survey and 771 second survey	1002 pregnant women for baseline survey and 771 mothers in the second survey	-
Yoshihiro Miyake, 2012 [18]	East	Japan	Japanese	Cross-sectional	April 2007 to March 2008	1741 pregnant women	-	-
Sachiko Inoue, 2013 [19]	East	Japan	Japanese	Cross-sectional	1997 to 2010	15,020 participants	-	Maternal Age ≤ 25: 1806 Maternal Age 25–35: 15,447 Maternal Age ≥ 35: 2416
Panchalli Wang, 2013 [20]	East	Taiwan	Taiwanese	Cohort	February 2010 to October 2011	198 women	-	Range 19 to 42
Su-Ying Tsai, 2013 [21]	East	Taiwan	Taiwanese	Cross-sectional	August 2011 to April 2012	715 mothers		Maternal Age 20–29:171 (23.9%) Maternal Age 30–39: 533 (74.6%) Maternal Age ≥ 40: 11 (1.5%)
Su-Ying Tsai, 2014 [22]	East	Taiwan	Taiwanese	Cross-sectional	August 2011 to April 2012	715 mothers	-	Maternal Age < 30: 23.9% Maternal Age ≥ 30: 76.1%
Su-Ying Tsai, 2014 [23]	East	Taiwan	Taiwanese	Cross-sectional	August 2011 to April 2012	608 mothers	-	Maternal Age 20–29: 139 (22.9%) Maternal Age ≥ 30: 469 (77.1%)
Dorothy Li Bai, 2015 [24]	East	Hong Kong	Hong Kong	Cohort	Cohort 1 (recruited in 2006–07) and cohort 2 (recruited in 2011–12). No substantial differences were found between cohorts	1738 women	-	Maternal Age 18–24: 4.5% Maternal Age 25- 29: 22.5% Maternal Age 30–34: 48.3% Maternal Age ≥ 35: 24.7%
Yuko Takayama, 2017 [25]	East	Japan	Japanese	Cross-sectional	-	158 nurses	-	Range 23 to 43
Yuka Yamazaki, 2017 [26]	East	Japan	Japanese	Cross-sectional	October 2012, over a period of 1 month	121 physicians	-	Age range of 20s: 4 (3.3%) Age range of 30s: 33 (27.35) Age range of 40s: 53 (43.8%) Age range of 50s: 23 (19.0%) Age range of 60s:7 (5.8%) No answer; 1 (0.8%)
Myung-Hui Kim, 2017 [27]	East	Korea	Korean	Cross-sectional	-	215 women	-	-
Jeong-Won Han, 2017 [28]	East	Korea	Korean	Cohort	-	625	Mothers of babies born in 2008	Mean age 32.74 for unemployed; 32.87 for employed
Farid Agushybana, 2018 [29]	Southeast	Indonesia	Indonesian	Cross-sectional	2012	1508	Data from the Indonesian Demographic Health Survey (IDHS) 2012	Mean 27.8 (standard deviation 6.3)
Zhuoyan Mao, 2018 [30]	East	China	Chinese	Cross-sectional	2015	247	-	25–29: 13% 30–34: 54% 35–39: 30% 40–44: 2% 45–49: 2%
Qun Wang, 2021 [31]	East	China	Chinese	Cross-sectional	March to December 2019	435 women	-	-
Su-Ying Tsai, 2022 [32]	East	Taiwan	Taiwanese	Cross-sectional	August 2011 to April 2012	715 mothers	-	Maternal Age 20–29: 99 (29.64%) Maternal Age ≥ 30: 235 (70.36%)
Mingxiao Liu, 2022 [33]	East	China	Chinese	Cross-sectional	January to November 2021	278	-	31 and above, most common (31%) in 31 to 32
Chunxiao Li, 2023 [34]	East	China	Chinese	Cohort	October 2017	216	-	Mean 29 (range 22 to 36)
Julan Xiao, 2023 [35]	East	China	Chinese	Cross-sectional	August 2020 to January 2021	2014	Pregnant women	Aged 18 to 34: 85% Aged 35 to 49: 15%
Fukuko Moriya, 2023 [36]	East	Japan	Japanese	Cross-sectional	February to March 2017 in Kurume; October and November 2017 in Saga	39	Physicians	Range 40 to 46
Akiko Kokubo, 2023 [37]	East	Japan	Japanese	Intervention	January to March 2018; November 2018 to March 2019	116	All were working	Mean 33 (standard deviation 3.5)

**Table 2:** Description of qualitative studies

Author, Year of Study	Region	Country	Ethnicity	Study Design	Study period	Sample size	Selection and specification of study sample	Age (range), years
Genaro Castro-Vázquez, 2015 [38]	East	Japan	Japanese	In-depth interview	-	Total of 27; 7 fulltime employees, 13 with part time jobs	19 of the 27 interviewees gave up work to get pregnant	Range 35 to 45
Ke Zhang, 2015 [39]	East	China	Chinese	Focus group and in-depth interview	-	50 mothers	-	Range 21 to 46
Heeyoung Han, 2018 [40]	East	Korea	Korea	In-depth interview	2015 to 2016	21	All women were working as physicians at the time of interview	30s to 50s
Yan Zhang, 2018 [41]	East	China	Chinese	Focus group and in-depth interview	2016	20 mothers who did individual interviews; 10 mothers who did focus group discussions	-	-
Nguyen Thi Truong Xuan, 2018 [42]	Southeast	Vietnam	Vietnamese	In-depth interview	March and April 2018	10	Working mothers either government officer or private officer, having breastfeeding experiences after return to work	-
Dzuriyatun Toyibah, 2019 [43]	Southeast	Indonesia	Indonesian	In-depth interview	-	15	-	-
Miliann Kang, 2020 [44]	East	Korea	Korean	In-depth interview	-	42	All women were working as teachers; 25 in public schools and 17 in private schools	-
Jiyoung Park, 2020 [45]	East	Korea	Korean	In-depth interview	August to September 2018	6	Working mothers living with private caregivers	Range 33 to 39
Yang Shen, 2020 [46]	East	China	Chinese	In-depth interview	2017 to 2019	26	Professionals with 2 children with at least 1 aged under 6 years	Mean 38, Range 30 to 46
Xin Bao, 2021 [47]	East	China	Chinese	In-depth interview + questionnaire	April to May 2021	12	-	Range 31 to 59
Ben Kerrane, 2022 [48]	Southeast	Singapore	Chinese	In-depth interview	2016 to 2017	10	Middle-class women who quite work to intensively parent their children	Range 31 to 45
Chompoonut Topothai, 2022 [49]	Southeast	Thailand	Thai	In-depth interview	2020	50	37 (74%) were employed at the time of the interview	Median age 31.5
Bunga A Paramashanti, 2022 [50]	Southeast	Indonesia	Indonesia	In-depth interview	2020	Total 46; 30 from Urban and 16 from rural	30% were working at the time of interview	Mean age 29.4 (standard deviation 6.71) for urban, 29.7 (5.92) rural
Li Bao, 2022 [51]	East	China	Chinese	Semi-structured interviews	-	6	These six participants were part of a larger study (n = 20). They were selected as they elaborated on the tension of motherhood and academic career	-
Boya Yuan, 2023 [52]	East	China	Chinese	In-depth interview	-	6	These six academic women who were from the disciplines of Humanities and Social Sciences (HSS) at six different non-elite public universities were selected as the research participants	Range 34 to 57

### Study methodological quality

**Fig. 2:**
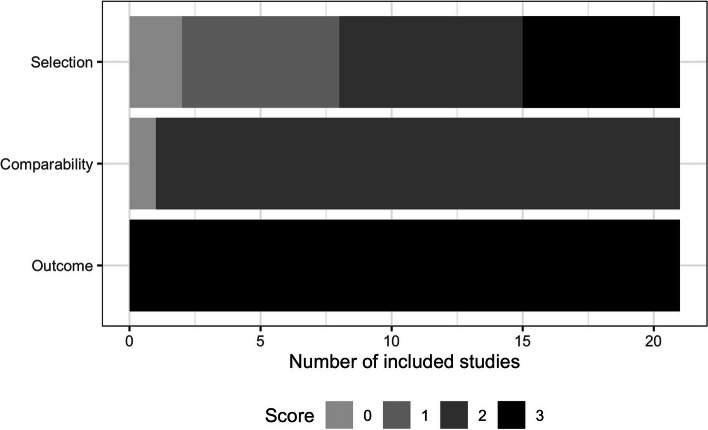
Graphical representation of quality assessment of included quantitative studies using Quality Assessment Tool (adapted version of Newcastle–Ottawa Quality Assessment Scale)

Among the 36 studies included in this analysis, one was an intervention study, 20 were classified as quantitative observational studies, and 15 were categorized as qualitative observational studies. Within the quantitative studies, 11 studies were rated as good and 9 studies were deemed satisfactory (Supplementary Table 2, and Fig. 2). It is worth noting that many of these studies did not provide a sample size calculation to justify their chosen sample size, which contributed to a lower quality rating. Additionally, a significant number of these studies depended significantly on participants' recall, which further impacted their rating. Regarding the qualitative studies, out of the 15 included, 6 studies did not mention obtaining ethics approval for their research (Supplementary Table 3).

### Study demographics

Most of the studies (*n* = 30, 83%) included in our analysis were carried out in East Asia (Tables 1 and 2), with the following distribution: 11 studies from China (5 quantitative and 6 qualitative), 1 study from Hong Kong (1 quantitative), 8 studies from Japan (6 quantitative, 1 qualitative, and 1 intervention), 5 studies from South Korea (1 quantitative and 4 qualitative), and 5 quantitative studies from Taiwan.

The remaining six studies were conducted in Southeast Asia: one cross-sectional study and two in-depth interviews were conducted Indonesia, and the remaining three in-depth interviews were conducted in Singapore, Thailand and Vietnam.

### Insights from 36 studies

We have synthesized findings from a total of five cohort studies [17, 20, 24, 28, 34], fifteen cross-sectional studies [18, 19, 21–23, 25–27, 29–33, 35, 36], two focus groups with subsequent in-depth interviews of a subset of women [39, 41], eleven in-depth interviews [38, 40, 42–46, 48–50, 52], one in-depth interview with structured questionnaire [47], one semi-structured interview [51], and one intervention workshop [37] (Supplementary Tables 4 and 5). It is worth noting that four of the cross-sectional studies conducted in Taiwan were derived from the same data source [21]. In total, the studies included a diverse population of 26,733 unique individuals.

We present an integrative review considering the variability in study design (i.e. in-depth interviews with non-aligned study aims and quantitative studies) and measure of outcomes. For example, breastfeeding (exclusive breastfeeding or breastfeeding with supplements from formula milk, depression (Edinburgh Postnatal Depression Scale (EPDS) or Center for Epidemiologic Studies Depression Scale (CED-D)), and employment (full-time only or included part-time work).

**Fig. 3:**
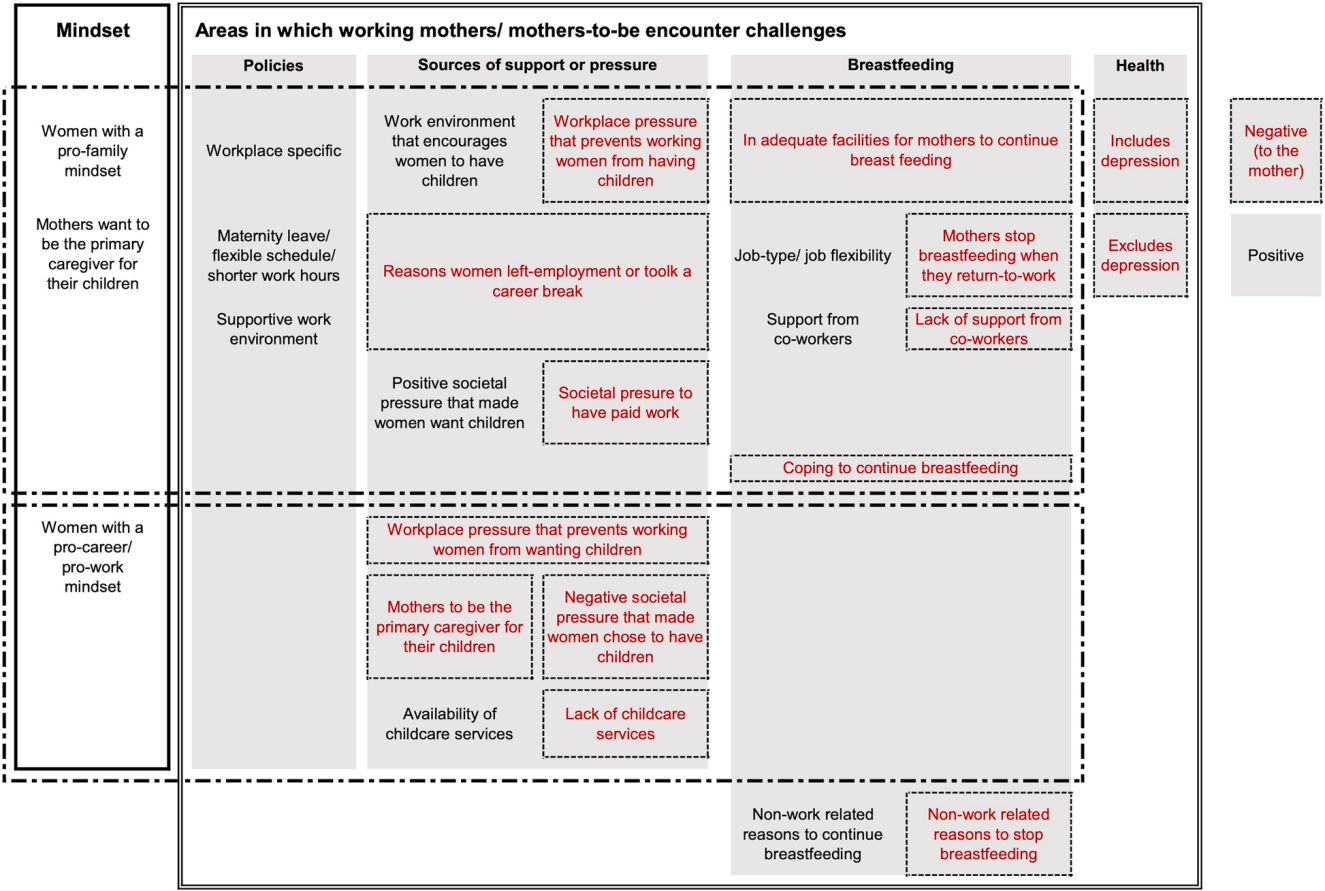
Grouping and clustering of themes

Post tabulation of the data, we grouped topics pertaining to challenges faced by working mothers and mothers-to-be into five themes 1) policies, 2) external support sources, 3) external pressure, 4) breastfeeding and 5) health status (Fig. 3). In addition, view-points of the mothers can be seen to differ with working mothers who have a pro-family mindset or a pro-career mindset (Fig. 3).

To facilitate our examination of the challenges encountered by working mothers, we commence with a concise overview of the employment landscape depicted in studies conducted in Asia, followed by an exploration of the specific challenges faced by mothers during the postpartum period.

#### Employment in Asia

In 2019, the female labour force participation across Asia, at 52.5%, is lower than that of males at 73.6%. (https://www.oecd-ilibrary.org/sites/2344a4f2-en/index.html?itemId=/content/component/2344a4f2-en#figure-d1e2369, accessed 18 March 2024). In particular, from highest to lowest of female labour force participation in countries included in this review: 72.7% in Vietnam, 61.9% in Singapore, 60.6% in China, 58.8% in Thailand, 53.8% in Indonesia, 53.8% in South Korea, 53.7% in Hong Kong, and 53.6% in Japan (https://www.oecd-ilibrary.org/sites/2344a4f2-en/index.html?itemId=/content/component/2344a4f2-en#figure-d1e2369, accessed 18 March 2024). Notably, countries with higher rates like Cambodia (76.9%) and Lao (76.5%), and countries with lower rates like Malaysia (51.3%) and Philippines (47.1%) were not included in our review. (https://www.oecd-ilibrary.org/sites/2344a4f2-en/index.html?itemId=/content/component/2344a4f2-en#figure-d1e2369, accessed 18 March 2024). The types of employment in these countries may differ from white-collared jobs (i.e. non-agricultural) described in the included studies.

#### Pro-work or pro-family – differences in mindset of mothers

The employment statistics does not show the number of mothers who chose to work or must work. The in-depth interviews showed that within every culture, there are some women with a pro-career mindset [26, 27, 34, 36, 40, 42–49, 51] and others with a pro-family mindset [27, 33, 34, 36, 38, 40, 42–44, 46–48, 51, 52].

Workplace policies like maternity leaves [44, 48], shorter work hours [44, 49], flexible schedule [43, 44, 47, 52] are valued by women with a pro-family mindset. Teaching and academic careers were both highly regarded as good career choices for pro-family mothers. Both careers had supportive work environment [43, 44, 47, 51, 52] not expressed in other industries (e.g. physicians [26, 40] and corporate jobs [38, 46]). A working mother (in academia) describes her choice to prioritize her child as “working hard for children and a better life gives life goals and motivation.” [47]. Another mother expressed her sacrifice of her career for childcare: “I know I have committed career suicide” [48].

Women with a pro-career/ pro-work mindset require more external sources of support, for example, better childcare services [45, 48], availability of hired help [40, 45, 46], and support from spouse and family [22, 40, 47, 51]. Instead of providing the mother time to care for the child (i.e. longer maternity leave, flexible schedule, or shorter working time), they need help to care for the child while they focus on their work. One mother used the word “privilege” to describe her younger colleagues requesting for different treatment: "I would recommend not requesting a privilege as a woman but working harder than male physicians in order to succeed." [40]. In the case where work done equates directly to income, priority was given to work: "I always think that breastfeeding is time-consuming … when I wish to finish my job and complete all the bakery orders. I earn a living from this work” [49].

The women interviewed expressed their understanding that it is difficult or impossible to achieve both a fast-paced career while being the primary caregiver for their child [43]. This is aptly expressed in “I think work–family balance is very important. However, it is a theory. In practice, it is impossible to balance work and family.” [40] and “If I can’t finish my work, I will take it home. I try my best to do everything, and I hope I can do it more perfectly.” [47].

#### Sources of support or pressure

When faced with similar situations, pro-family and pro-career mothers will react in different ways. In this section, we will highlight external pressures faced by working mothers and support sources available to them, from their workplace [34, 38, 40, 42–44, 46, 48, 51] and from society [38, 40, 44, 48]. Specific to pro-family mothers, the reasons they left the job that they initially had prior to having a child include: taking a career break, or choosing work with more flexible schedules. For pro-career mothers, their concerns were mostly related to their career [26, 27, 40, 48].

##### Support sources are necessary for mothers to continue paid employment or focus on their career

Only one study in Japan specifically examined women who chose to leave their employment upon deciding to have children (Supplementary Table 5) [38]. A Singapore’s study, similarly, identified a group of mothers who left employment to focus on childcare (Supplementary Table 5) [48].

In Japan, married women are expected to become mothers. In addition, their society commonly believes that "a mother is still seen as a complete woman" [38]. Childless married women faced the need to provide explanations for their circumstances, and a sense of alienation from their peer groups [38]. Additionally, their workplace perceived managerial or supervisory roles as incompatible with motherhood. The women who chose to fulfil their instinctual desire to become mothers or conform to traditional norms relinquished their careers to fulfil their instinctual desire to become mothers or conform to traditional norms.

Conversely in Singapore, the mothers were questioned: “why are you wasting your education?” [48]. Educated women are expected to take on paid work.

While the mothers from both studies live in societies that are vastly different, similarities can be observed – 1) they were from affluent households, 2) they are of the pro-family mindset, and 3) they faced unsympathetic colleagues and superiors when they were in paid employment. With financial stability, their circumstances provided them with a choice to focus on having and raising children without the worry of finance. This may not be the same for women from less financially affluent households.

Should they choose to continue working, access to trustworthy childcare services is needed so that they can focus on their work (Supplementary Table 5) [45, 48]. Grandparents were the most mentioned support received by working mothers [40, 46, 47, 51]. Male spouses that were willing to go against the East Asian norm and be the more involved parent, were another supportive factor for career focused mothers [45–47, 51]. Lacking access to quality childcare services resulted in stress for the mother, mothers opting for more flexible jobs, or leaving paid employment [45, 48].

Mothers who took a career break recognize that it would be difficult to return to work [48]. However, it is believed by pro-family mothers that their role in childcare cannot be replaced by paid help or other family members (Supplementary Table 4 and 5) [46, 48]. In a different study, re-integration as a potential solution was mentioned [44]. Collectively, Korean teachers helped those who took career breaks to re-integrate back to the workplace [44]. Re-integration was also studied in an intervention study, through a four sessions workshop that aims at improving the mother’s self-efficacies [37].

Rather than re-integrating, some working mothers may benefit more from a supportive work environment that allows them to continue working. Three studies have emphasized the connection between a supportive work environment and various aspects of work, including satisfaction, identity, and conflict (Supplementary Table 4) [26, 27]. In Japan, physician mothers reported higher career satisfaction when perceiving a better work-life balance compared to those without children [26]. However, a greater number of physician mothers expressed reconsideration of their full-time clinic practice [26].

##### Workplace stressors

Gender inequality posed obstacles to women's job opportunities, job security, and career advancement (Supplementary Table 5) [38, 40, 44, 46, 48, 51]. Interviewees emphasized “the identity of women has a negative impact on academic career development” [51] and “Plus, women have fewer opportunities. We have about 30 section chiefs (ke zhang), but only one of them is female” [46]. Even in sectors dominated by females, males were more likely to receive promotions compared to their female counterparts [44]. Korean female physicians expressed concerns about pregnancy, as an unspoken rule suggested that single women should not become pregnant during their residency [40]. Similarly, in Singapore, women expressed concerns that maternity leave could impact their chances of promotion [48].

Being discriminated against for career progression acted as encouragement for some working mothers to focus on family building [46]. However, gender discrimination is not synonymous to discrimination against mothers. Discrimination targeted at females with children will result in mothers leaving the workplace [38, 46, 48].

Two studies discussed solutions that address workplace stressors through interventions that improve self-efficacy of mothers (Supplementary Table 4 and 5): 1) job crafting [34] and 2) re-integration to work workshop [37]. Job crafting was studied in three categories, task, relational and cognitive crafting. All three measures had positive impact on the mother’s work-to-family enrichment, i.e. the mother’s work improved her self-efficacy and benefited her role in her family [34]. The re-integration workshop showed that improvement in work-family balance and self-efficacy resulted in better in-role performance at work but not on work-attitudes [37].

#### Challenges of breastfeeding

Breastfeeding emerged as the most frequently discussed topic across the included studies (n = 13 of 36 publications (Supplementary Table 4 and 5) [17, 21–24, 29, 30, 32, 39, 41–43, 47, 49, 50]). In addition, breastfeeding is commonly the first physical challenge faced by mothers upon returning to the workplace.

Breastfeeding has the most specific challenges, where solutions proposed by some studies were observed to work in others. In general, challenges for working mothers to continue breastfeeding can be classified into 1) inadequate facilities, 2) lack of job flexibility, 3) lack of support from co-workers, 4) non-work-related reasons.

Interviewees from studies conducted in countries with varied cultures highlighted similar inadequate facilities [30, 39, 41, 42, 49]. For example, “There was no refrigerator and no private space in the office” from the Thai study [49], “There is no private room for pumping milk at my workplace” from a Chinese study [39], and “The refrigerator there…but it is not safe for keep the milk…because for keeping the milk we need a really clean suitable temperature referent” from a Vietnamese study [42]. Providing facilities like a private room with a refrigerator dedicate for breast milk storage have been shown to decrease early cessation of breastfeeding [30]. One interviewee included provision of breast pumps at the workplace as part of the facilities. Unlike the private room solution, provision of breast pumps has not been mentioned by other studies [42]. However, this likely would help mothers who faced inconvenience in travelling to work via crowded public transports [41].

Working in an office is better than working in a factory if adequate facilities to express breast milk are available. Combined with flexible schedule seen in countries like Indonesia and Korea, for example, in the teaching industry and agriculture, mothers have the option of continuing to breastfeed. On the other hand, clean room employees or shift work employees do not have a conducive environment to continue breastfeeding. Unlike the issue with inadequate facilities, job type/ job flexibility is difficult to adjust for. Wanting to continue breastfeeding, yet faced with inflexible job schedule, mothers change their jobs or quit employment. These pro-family mothers are willing to sacrifice their careers for their child.

Established workplace policies which promotes breastfeeding can enable returning mothers to continue breastfeeding with the support from their colleagues [21]. In a Taiwanese manufacturing factory, returning mothers who were aware of the provision of time-off and lactation room to express breastmilk and were working in the office environment were more likely to continue breastfeeding as compared to mothers working in the clean room setting. However, mothers who took the time-off to express breastmilk were conscious that they may be seen as less productive [23]. This sentiment is also expressed by an interviewee from China “When I went to express milk, my boss sometimes was uncomfortable with it, because it took half an hour every time. It seemed that I left my seat while I went to express milk. And some colleagues reported to the boss. …. My boss is male, and he is not empathetic!” [41]. The lack of support from colleague was explicitly mentioned by a Vietnamese “I could not receive any support from my office” [42]. Having policies in place is insufficient, and colleagues and supervisors’ encouragement to utilize the provided time-off is necessary for mothers to be comfortable in continuing breastfeeding [21–23].

The challenges faced by working mothers in continuing breastfeeding is not limited by the workplace [32, 41, 49, 50]. Conversely, there are non-work-related factors that encourages working mothers to continue breastfeeding [29, 39, 42, 49, 50]. Some working mothers have developed coping strategies to continue breastfeeding even in not conducive workplaces [41, 49, 50].

#### Health status and the impact on working mothers

Employed mothers exhibited better mental and social health statuses compared to their non-employed counterparts [20]. These findings align with two studies conducted in Japan, which also highlighted the relationship between employment and reduced rates of depression among mothers [17, 18].

Depression is the most studied health status, with three studies utilising the Edinburgh Postnatal Depression Scale (EPDS) [17, 31, 35], one using the Center for Epidemiologic Studies Depression Scale (CES-D) [18], and another using a questionnaire developed by Kessler et al. [28]. In Japan, it was observed that employed women had lower levels of depressive symptoms, as measured by the EPDS or CES-D, compared to unemployed women [17, 18]. This was also observed in the Chinese study where unemployed women expressed higher depression than employed (odds ratio [95% Confidence Interval]: 1.53 [1.21 to 1.94], *p* < 0.001) [35].

Employed mothers with young children may face the risk of experiencing burnout syndrome. Burnout syndrome was examined in Japan using the Maslach Burnout Inventory scale (MBI) [25]. Mothers (nurses) who did not have a supportive work environment, and worked long hours of overtime were more prone to experiencing burnout [25]. Additionally, mothers with higher MBI scores were more likely to express a desire to resign or seek a change in their job responsibilities [25]. Related to burnout syndrome, a Chinese interviewee explains that she has to do the household chores, on top of taking care of her children and felt “very tired in such a recurring life” [47]. It is not uncommon that mothers neglect their health to fulfil their role as a mother [42, 47]. Having access to external support would allow mothers to have time to take care of their own well-being [45].

Mothers may feel stress at various stages, antenatal or when their children are young [35, 47]. Perceived stress was associated with increased depression [28, 35]. It was shown in a separate study that working mothers with younger children perceived higher stress than those with older children [33]. Feeling an emotional sense of parenting effectiveness was associated with lower stress [33]. More studies in various job types, are needed to develop re-integration plans for mothers returning from maternity leave [37, 44, 48].

## Discussion

Our systematic review explored the pro-career and pro-family mindsets observed in working mothers from East and Southeast Asia. Overall, our findings build on the limited research that suggest the different mindsets working mothers have, which lead to different immediate priorities and choices, contrasting challenges faced, and consequently requiring different extents of support. Pro-family working mothers often find that their responsibility for childcare is irreplaceable, even with the availability of paid childcare services or help from family members. They therefore tend to leave the workplace or choose careers with flexible schedules to focus on their children. On the other hand, working mothers with a pro-work mindset outsource childcare needs.

It is important to acknowledge that some choices can only be made with the privilege of resources and stable financial capabilities. Working mothers who can afford to leave the workforce to focus on their children might come from a relatively financially stable background. This supports the current body of literature that explores the manifestation of gender roles in parenthood. Working mothers prioritising childcare may also benefit from having high-earning partners [48, 53, 54]. The phenomenon of "marrying up," which refers to the prevalent preference for marrying into a higher social or economic status, and the practice of treating their child as a "little emperor" by focusing solely on them, are more commonly observed in Asian countries [55, 56]. These cultural factors reinforce traditional gender roles, where men are expected to be the primary breadwinners and women are assigned the role of homemakers [57–60].

With the need for financial stability, equipping a pro-family mindset seems to be a luxury that some working mothers cannot afford. The dynamic [61] of dual-income parents has become increasingly common due to rising living costs. Especially in Singapore, parents signing up their children for tuition and supplementary classes is the societal [62] norm. Parental affection in some families has evolved from being interpersonal with their children to perfecting their children’s future through intensive parenting. This highlights that pro-career working mothers do not necessarily love their children less than pro-family working mothers, as the reasons behind their choices are multifaceted and nuanced, influenced by Asian family sentiments, financial capabilities, resources available and societal pressures. It is important, however, to note that personal and societal values of pro-family and pro-career mindsets are not set in stone. This adds to the complexity of balancing work and family, since the variables affecting mindsets are fluid. Consequently, some mothers who chose to forgo their careers had regrets [26, 47] and some mothers who focused on their careers and felt guilty for neglecting their children when they were young [47, 52].

Having examined the reasons behind certain choices and mindsets that working mothers take on, the collective impact of these decisions needs to be considered as well. In Asian countries such as Japan, Korea, and Taiwan, there is a significant correlation between the educational attainment of husbands and wives [63]. While the decision to voluntarily leave the workforce may appear acceptable on the surface, societal norms that perpetuate gender biases hinder progress toward achieving gender equality in the workplace.

Working mothers encounter workplace discrimination that is further exacerbated by stereotypes and traditional gender roles. Employers, whether consciously or unconsciously, often question the commitment of women who become pregnant or have young children, leading to unemployment through contract non-renewals or terminations [26, 44, 64]. It's important to note that discrimination against pregnant women and mothers is not limited to male or childless employers [64]. To unlock the potential of all employees, it is crucial to create an environment that allows women to fulfill their maternal responsibilities without permanently derailing their career paths.

Without resolving the gender inequality, over-emphasis on maternal rights may backfire and result in lesser opportunities given to females of childbearing age. Even in the most prosperous nations, gender equality remains elusive [65]. The tendency for peer comparison leads to social anxiety and increased dissatisfaction [66]. To address this, women may need to reassess their career aspirations and consider alternative paths that deviate from traditional promotions and age-based milestones [67]. When it comes to hybrid job opportunities, working mothers must navigate the negotiation of flexible work arrangements without it being perceived as a trade-off for their professional contributions [68]. Fortunately, advancements in technology have provided tools to redefine job roles, enabling companies and employees to collaborate and find a mutually beneficial approach that maximizes human potential.

While technology advances, societal values and gender roles similarly evolve as well. The responsibility of childcare is no longer wholly of the mother, but also of the father. Adjusting to these systematic, undercurrent views, changes must be made to policies for them to be effective. Mandatory policies like maternity and paternity leaves are in place in various countries, e.g. China, Korea, and Singapore [69]. However, the length of paternity leave is not on par with maternity leave. Polices, like maternity leaves, flexible schedules, and shorter work hours benefit working mothers with a pro-family mindset. These mothers want to spend more time with their children and be the primary caregivers for their children [40, 48]. On the other hand, these policies are not conducive for mothers with a pro-career mindset. They need more external support (e.g. paternity leave) to mind their child while they focus on work. Assuming all mothers want to work less and having societal norms that childcare is a mother’s role harms the career prospects of pro-career mothers [44, 51].

Several initiatives have been deployed in the workplace to help working mothers. Wellness programmes implemented to improve women’s health have been documented in the United States, the United Kingdom and Thailand [70]. However, the studies examined in this systematic review did not mention such initiatives. There are limitations on what a company can do at the ground level [71] – adding facilities to allow the expression of breast milk in the office [72–74], educating staff to encourage breastfeeding and being tolerant of childcare needs [21, 23], and increasing maternity leaves [44, 75, 76]. However, there are drawbacks to implementations targeted at the female population. Emphasizing the increased need for mothers to have time for breastfeeding or childcare may be viewed as reduced productivity [23]. When males are more present in the office, an unconscious bias against females may occur [77].

On a national level, many countries have implemented measures such as paid maternity leave and lactation rooms to support breastfeeding when mothers return to work [78]. However, these initiatives alone do not address the root issue of gender discrimination. By placing the primary caregiver role solely on females and neglecting equal paternity leave for fathers, and by emphasizing time off for breastfeeding, we inadvertently contribute to lower productivity among women and perpetuate gender discrimination. To combat these biases, it is important to promote equality in paternity leave and enhance fathers' ability to take on caregiving responsibilities. While no solution is perfect, countries like Finland and Norway have made notable progress in promoting gender equality in the workplace [65]. Asian countries, with lower Gender Gap indexes and none reaching 0.8 (where 1 represents gender equality), have room for improvement [65]. However, despite the appeal of paternity leave in theory, its uptake might be limited, particularly in Asian populations, due to its misalignment with current gender norms [79].

Regardless of whether working mothers lean towards pro-work or pro-family mindsets, it is important to ensure that being a mother does not discriminate between those who can afford it and those who cannot. The need for a supportive work environment is therefore recognized. Despite this, implementation remains challenging. Currently, the most tangible steps involve providing physical facilities like lactation rooms and implementing policies that encourage breastfeeding for the initial postpartum period [72–74]. Addressing issues of gender inequality and missed opportunities in the workplace requires systemic changes that are difficult to achieve at the company level [40, 44, 64, 75, 76, 80–82]. By drawing inspiration from societies like Finland and Sweden, where gender equality is more prevalent, we can learn valuable lessons and strive for a less discriminatory work environment [83]. Furthermore, shifting our mindset to recognize that success goes beyond job status and monetary value is not only beneficial for working mothers but also for society [84].

Due to the diversity in variables measured and statistical analyses used across the quantitative studies, conducting a meta-analysis was not feasible. As a result, the comparability of the included studies is limited. Furthermore, several cross-sectional studies did not adequately justify their sample sizes and relied solely on parental recall, which led to a lower score when assessed using the adapted version of the Newcastle–Ottawa Quality Assessment Scale.

## Conclusion

Employment contributes to improved mother’s well-being. Nevertheless, if work environments are restrictive or workloads become overwhelming, it can result in burnout and lead mothers to leave their jobs. Mothers of pro-family and pro-career mindsets require different supports. The most obvious being longer maternity leaves, flexible job schedule, and shorter work hours for pro-family mothers; pro-career mothers would prefer better access to quality childcare and support from spouses. While the gender gap in education is narrowing, there is still a need to address the economic impact caused by women leaving the workforce due to motherhood. It emphasizes the importance of conducting more rigorous and comprehensive studies to generate valuable insights for informing diversity policies across different workplaces.

### Supplementary Information

Supplementary Material 1.

## Data Availability

No dataset was used for this systematic review. All information used are from published sources listed in Tables 1 and 2.
